# Characterizing Indoor Black Carbon Dynamics in a Residential Environment: The Role of Human Activity and Ventilation Behavior

**DOI:** 10.3390/toxics13070536

**Published:** 2025-06-26

**Authors:** Nikolina Račić, Sanja Frka, Ana Cvitešić Kušan, Valentino Petrić, Francesco Mureddu, Mario Lovrić

**Affiliations:** 1 Institute for Medical Research and Occupational Health, 10000 Zagreb, Croatia; nracic@imi.hr; 2 The Lisbon Council, 1040 Brussels, Belgium; valentino@ascalia.io (V.P.); francesco.mureddu@lisboncouncil.net (F.M.); 3 Ruđer Bošković Institute, 10000 Zagreb, Croatia; frka@irb.hr (S.F.); ana.cvitesic.kusan@irb.hr (A.C.K.); 4 Ascalia d.o.o., 40000 Cakovec, Croatia; 5 Institute for Anthropological Research, 10000 Zagreb, Croatia; 6 Faculty of Food Technology Osijek, Josip Juraj Strossmayer University of Osijek, 31000 Osijek, Croatia

**Keywords:** air pollution, biomass burning, concentrations, fossil fuel, homes

## Abstract

Understanding indoor black carbon (BC) dynamics is important for assessing human exposure and informing air quality management in residential settings. This study presents a high-resolution, multi-sensor dataset collected over 24 days in a semi-occupied home in Zagreb, Croatia, designed to characterize the temporal behavior and sources of indoor BC. Indoor BC concentrations were measured at 1 min resolution using a dual-spot aethalometer, with source apportionment into biomass burning and fossil fuel components. Complementary contextual data including motion detection, door and window states, and traffic activity were collected in parallel using smart sensors and annotated experimental logs. Across the monitoring period, daily mean BC concentrations ranged from 174.7 and 1053.1 ng/m^3^ for biomass burning BC and between 53.2 and 880.3 ng/m^3^ for fossil fuel component. Statistical analyses revealed significant increases in BC concentrations during direct combustion-related activities, including scented candle burning and gas burner use. Additional BC elevations were associated with mechanical heat sources and nearby vehicle traffic, particularly affecting the fossil fuel BC component. In contrast, non-combustion activities such as brief human presence exhibited minor or inconsistent effects on indoor BC levels. This study elucidates the primary role of combustion-based indoor activities in influencing short-term BC exposure and highlights the importance of synchronized, high-resolution datasets for indoor air quality research.

## 1. Introduction

Air pollution remains one of the leading environmental health risks worldwide, contributing to an estimated 7 million premature deaths annually [1,2,3,4]. Among the different air pollutants, particulate matter (PM) is one of the most important contributors to health outcomes, with extensive epidemiological and toxicological evidence for both short- and long-term exposure to increased risks of cardiovascular and respiratory diseases, pulmonary inflammation, lung cancer, and neurodegenerative and developmental disorders [5,6,7,8,9,10]. Within the fine particulate matter fraction, black carbon (BC)—a component of PM and resulting dominantly from incomplete combustion of fossil fuels, biomass, and other carbon-rich materials—has gained increasing attention due to its dual impact on human health and climate change [11,12,13,14,15,16]. BC is particularly harmful because of its small particle size, high surface area, and ability to adsorb and transport toxic organic compounds [17]. Exposure to BC has been associated with systemic inflammation, oxidative stress, impaired lung function, and increased risk of cardiovascular morbidity [18,19]. Vulnerable populations, such as children, the elderly, and individuals with pre-existing health conditions, are especially at risk [20,21,22]. Many research initiatives, including the Horizon Europe EDIAQI project (Evidence-Driven Indoor Air Quality Improvement, https://ediaqi.eu/; Grant Agreement No. 101057497), are actively investigating the impacts of indoor air pollution on human health, with a particular focus on vulnerable populations and evidence-based strategies for improving indoor air quality [23,24]. Importantly, indoor exposure can account for a significant amount of an individual’s total BC exposure, particularly in regions or seasons when people spend most of their time indoors. Despite growing evidence on outdoor BC levels and their impacts, indoor concentrations remain understudied, particularly in real-life residential settings. Indoor BC levels can be influenced by both outdoor infiltration and indoor sources, such as cooking, heating, and the use of candles or other combustion-based products [25,26,27]. Outdoor sources of BC primarily stem from incomplete combustion of fossil fuels and biomass, including vehicular traffic, residential wood burning, industrial activities, and, in some regions, open agricultural burning [28,29,30]. In addition, human behavior (e.g., window opening, occupancy patterns) and ventilation dynamics have an impact on modulating indoor concentrations [31,32,33].

This study presents a high-resolution dataset focused on indoor BC dynamics in a residential setting. By integrating BC measurements with detailed contextual information, including human activity, ventilation behavior, and pollution sources, this work aims to provide a better understanding of the drivers of indoor BC variability and to support improved exposure assessment in everyday environments.

To our knowledge, this is the first study to measure indoor BC concentrations in a residential setting in this region using both high-resolution source-apportioned BC data and an extensive network of supporting sensors. While previous research in Croatia and the broader region has primarily focused on ambient air pollution and outdoor PM, comprehensive indoor monitoring, particularly with synchronized data on human activity, ventilation, meteorology, and source events, is lacking. This dataset and study design provide a rare opportunity to explore how indoor BC levels fluctuate in response to real-life behaviors and environmental conditions, contributing a better understanding of both regional air quality research and broader efforts to assess indoor pollutant exposure.

## 2. Materials and Methods

### 2.1. Experimental Setup and Instrumentation

**Figure 1:**
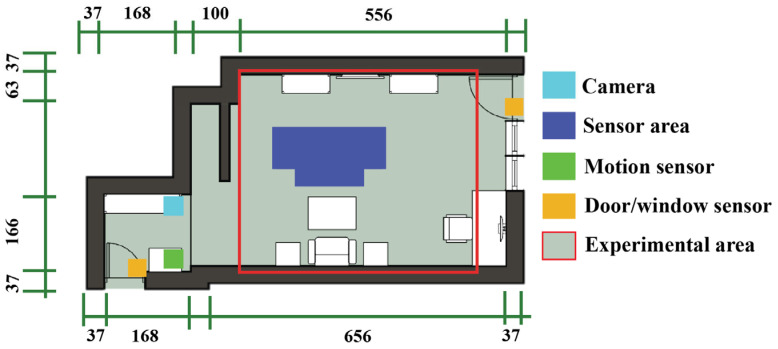
Experimental room layout and sensor placement in residential monitoring setup (measured in cm).

BC concentrations were measured over a 24-day period, from 11 October to 3 November 2024, in a residential setting in Zagreb, Croatia (Figure 1). A BC monitor was used indoors to continuously capture high-resolution measurements of total black carbon, with source apportionment distinguishing between biomass burning and fossil fuel combustion, reported in nanograms per cubic meter (ng/m^3^). BC was measured using a dual-spot aethalometer (AE33, Magee Scientific, Portland, OR, USA), which records aerosol light absorption at seven wavelengths (370–950 nm). The instrument operated at a 1 min time resolution and a flow rate of 5 L/min, with online correction for filter loading via dual-spot technology. BC concentrations were calculated from attenuation at 880 nm using a mass absorption cross-section (MAC) of 7.77 m^2^/g and a multiple scattering parameter of 1.39 [34].

To understand the drivers of BC variability, additional environmental and behavioral data were collected in parallel, which are described in an indoor and ambient air pollution dataset using a multi-instrument approach and total event monitoring [35]. Human presence was monitored using a motion sensor and a camera, while the states of windows and doors were recorded using Xiaomi smart sensors (Beijing, China) installed at key access points. These contextual data allowed for the detection of main indoor emission events such as candle burning or occupancy-related ventilation. All indoor activities and experimental interventions were timestamped and recorded in a structured file, enabling precise temporal alignment between observed BC peaks and potential emission or ventilation events.

In addition to continuous monitoring, the study employed a structured experimental design to simulate everyday household activities known to influence indoor air quality. A total of 25 distinct events were conducted during the 24-day campaign, including activities such as candle burning, cooking (gas burner), diffuser use, vacuum cleaning, human presence, physical exercise, and heating via electric or central systems. Ventilation scenarios were also tested through controlled opening of doors to the garden and hallway, simulating infiltration of outdoor air under different conditions. Each event was carefully timestamped and annotated, recording the exact start and end times, event type, and conditions (e.g., doors open or closed). This enabled the alignment of high-resolution BC concentration data with specific indoor activities and environmental changes. The baseline period before each event was left undisturbed, and in most cases, a 15 min stabilization period preceded each new intervention to ensure comparability across experiments.

This setup enabled the differentiation between background indoor concentrations and activity-induced peaks, allowing for the assessment of event-driven variability in BC concentrations with minute-level precision. The reproducible structure of these interventions also facilitates statistical comparison between before, during, and after periods, supporting robust analysis of the impact of specific behaviors on indoor BC levels.

### 2.2. Statistical Analysis

To enable temporal alignment and comparison, data from motion detection, sensors, traffic recordings, and environmental monitoring were resampled or transformed into minute-level binary flags or averaged where appropriate. For each identified indoor activity or intervention (e.g., candle burning, cooking, diffuser use), BC concentrations were segmented into three time periods: before, during, and after the event. The event windows were defined based on a structured JSON log, with 10 min windows typically used for the before and after periods. Events were treated as independent, non-overlapping interventions. A short period on 30 October (11:20–11:22) was excluded from the analysis due to abnormally high BC readings (>8000 ng/m^3^) in the absence of any logged activity. This was considered a measurement artifact, likely linked to instrument initialization. To assess whether BC concentrations differed significantly across these periods, two complementary statistical tests were applied. One-way analysis of variance (ANOVA) was used to test for differences in mean BC concentrations between the three periods, assuming normality and equal variance. The Kruskal–Wallis H test, a non-parametric alternative, was used to validate results without assuming normal distribution of the data. *p*-values from both tests were reported for each event, and statistical significance was evaluated at α = 0.05. Events with significant *p*-values in either test were considered to have a measurable effect on BC concentrations.

In addition to event-based analysis, summary statistics (mean, minimum, maximum) of BC concentrations were calculated daily, and BC levels were also compared between daytime, nighttime, and late night periods to examine diurnal trends. Time of day segments were defined as follows: day: 07:00–22:59; night: 23:00–02:59; late night: 03:00–06:59. All analyses were conducted using Python (v3.10) with the pandas (v2.2.2), numpy (v1.26.4), scipy (v1.13.1), and matplotlib (v3.9.2) libraries. Generative AI tools were used during the preparation of this manuscript to support the review and structuring of Python code used in the analysis. Specifically, ChatGPT (Open-AI, GPT-4) was employed to improve clarity of language, check code logic, and suggest efficient implementation approaches. All outputs were critically evaluated, edited, and verified by the authors to ensure accuracy and scientific validity.

## 3. Results and Discussion

### 3.1. General Black Carbon Concentration Levels and Temporal Variability

During the 24-day monitoring campaign, indoor black carbon (BC) concentrations demonstrated notable day-to-day and hourly variability. Summary statistics of daily BC concentrations, including daily mean, minimum, and maximum values, are presented in Table 1.

Daily mean concentrations of total BC (biomass + fossil fuel) ranged from 356.62 ng/m^3^ on 13 October to a maximum of 1933.37 ng/m^3^ on 30 October. The highest single-minute BC concentration recorded was 5430 ng/m^3^, observed during a peak event on 21 October. Several days exceeded a mean around 1000 ng/m^3^, including 21 October (1347.43 ng/m^3^), 24 October (1009.46 ng/m^3^), 29 October (1111.63 ng/m^3^), 30 October (1943.82 ng/m^3^), and 31 October (1547.11 ng/m^3^), suggesting the occurrence of major indoor or nearby outdoor emission events. The highest biomass burning BC mean was observed on 30 October (1053.09 ng/m^3^), while the highest fossil fuel BC mean value occurred on the same day (880.28 ng/m^3^), suggesting that this day likely involved both indoor and infiltrated outdoor combustion sources.

**Figure 2:**
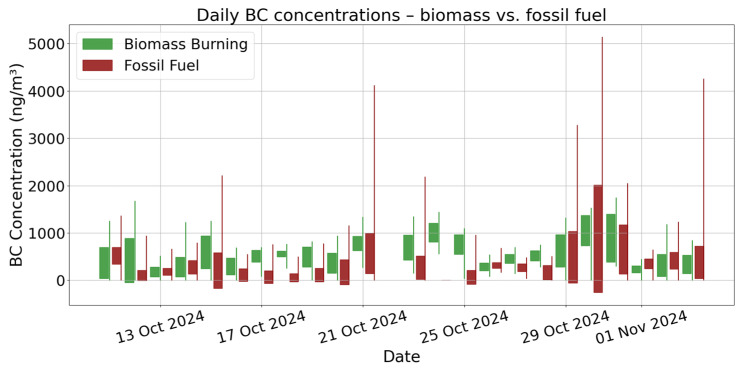
Time series of BC concentrations from biomass burning and fossil fuel combustion over 24-day indoor monitoring period. Each bar represents daily mean ± standard deviation and full min–max range of indoor BC levels in residential setting in Zagreb, Croatia.

Temporal variability and relative contributions of biomass and fossil fuel combustion to indoor BC levels are presented in Figure 2. Biomass-related BC exhibits more sustained elevations over more extended periods while, in contrast, fossil fuel BC shows sharper, transient peaks. Notably, several major BC peaks in the final third of the campaign are dominated by fossil fuel signals, explaining the importance of outdoor influences. When analyzed by time of day (Table 2), BC levels were typically higher during late night and nighttime periods. Total BC levels were highest at night (898.71 ng/m^3^), followed by late night (786.25 ng/m^3^), and lowest during the day (775.72 ng/m^3^). Biomass burning BC showed a clear increase outside of daytime, with the highest mean during the night (698.43 ng/m^3^) and late night (662.22 ng/m^3^), compared to 441.73 ng/m^3^ during the day. In contrast, fossil fuel BC exhibited a different relation, with higher daytime concentrations (333.60 ng/m^3^) and lower levels during late night (124.02 ng/m^3^). These results suggest that indoor sources such as biomass-related activities may dominate during periods of reduced ventilation and activity (night and late night), while fossil fuel-related BC may be more influenced by daytime outdoor infiltration or human-related activity patterns. These diurnal patterns, combined with sharp episodic spikes, explain the importance of high-resolution measurements to capture both background levels and short-term fluctuations.

### 3.2. Impact of Indoor Activities on Black Carbon Concentrations

To assess how everyday household activities influence indoor BC concentrations, 25 controlled experiments were conducted over the monitoring period. Each experiment simulated a specific real-life scenario including combustion sources (e.g., candles, gas stove), human activity, ventilation, and external sources (e.g., traffic, burning).

**Figure 3:**
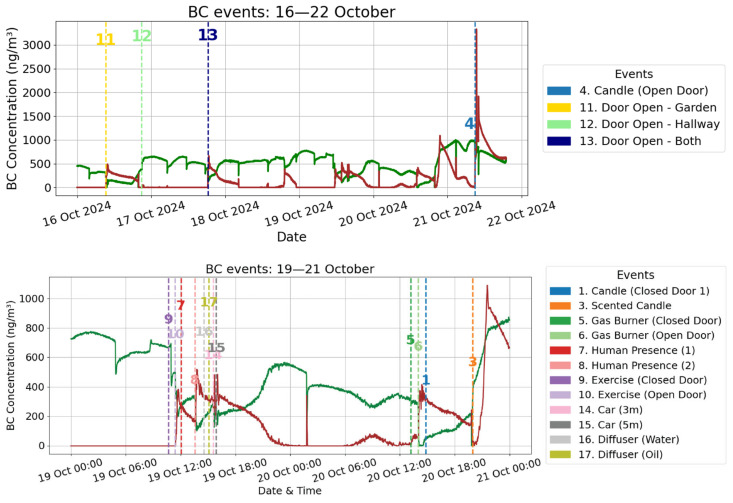

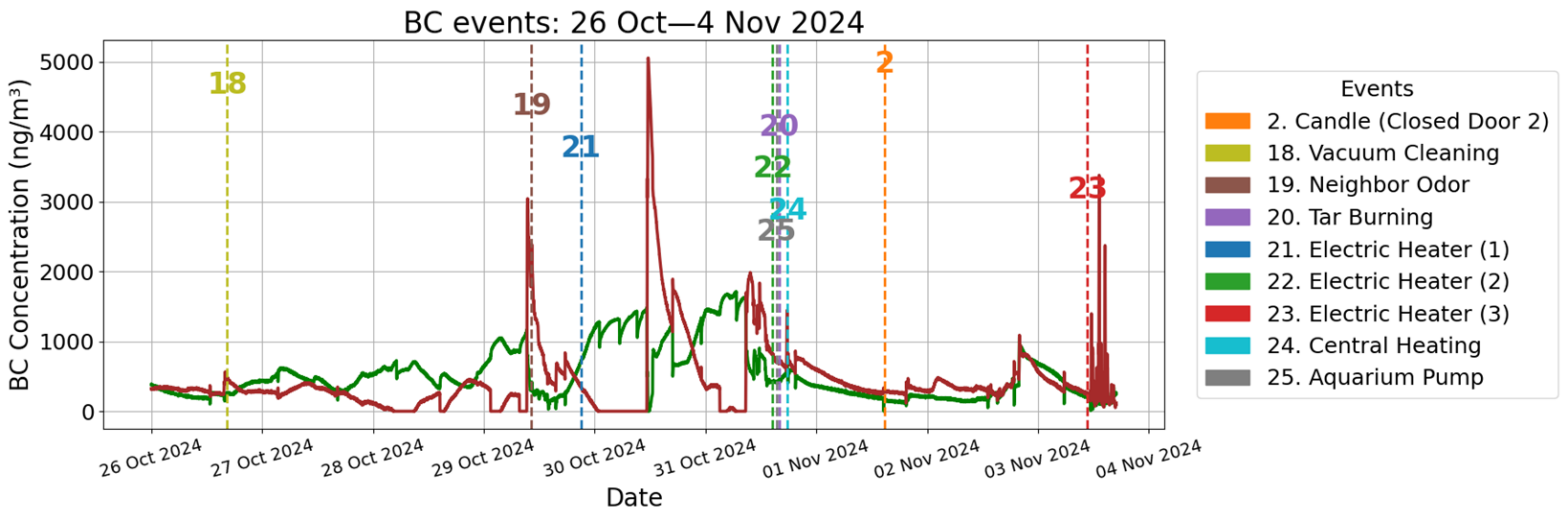
Temporal BC concentration (biomass burning—green; fossil fuel—red) with 25 experimental events.

Figure 3 shows the complete 24-day time series BC concentrations, with vertical dashed lines marking the timing of 25 experimental and real-life indoor events. The selected events encompass a diverse range of emission sources and activities, including combustion (e.g., scented candles, gas burners, radiator heating), human presence, ventilation behavior, and other everyday household actions (e.g., vacuum cleaning, aquarium pumps). Each event is labeled numerically on the plot and cross-referenced with the legend on the right.

Visual examination of the BC concentration time series reveals that several pronounced peaks are closely aligned with specific combustion-related activities, particularly the burning of a scented candle, operation of an electric heater, and activation of the central heating radiator. These events are among the highest peaks observed in the dataset, clearly indicating their substantial contribution to short-term increases in indoor BC concentrations. The elevated levels during and following these activities are consistent with known emission profiles of incomplete combustion and heat-generating devices, which are familiar sources of indoor BC. Events 11 (door Open—garden) and 13 (Doors Open—Garden + Hallway) were followed by moderate increases in BC from biomass burning, suggesting that these ventilation actions may have facilitated the infiltration of outdoor air polluted by nearby residential combustion and local traffic sources. In contrast, event 12 (Door Open—Hallway) did not lead to a noticeable BC increase, likely because the hallway served as an indoor buffer zone rather than an outdoor air pathway. Event 4 (Candle Burning—Open Door), occurring on 21 October, resulted in a pronounced spike in biomass-related BC, reflecting the strong impact of indoor combustion when doors are open and smoke can circulate more freely. These results illustrate how both external infiltration and indoor activities contribute to temporal variability in indoor BC, with indoor sources exerting a more immediate and substantial impact. Physical activity (events 9 and 10) had no discernible effect on BC levels, indicating that exercise alone does not generate or resuspend measurable BC particles. In contrast, events related to combustion and vehicle activity triggered apparent concentration shifts. Cars idling near the building (events 14 and 15—3 m and 5 m distance) corresponded with sharp increases in fossil fuel-related BC, confirming traffic emissions as an important external source of infiltration. Gas burner use caused noticeable spikes in fossil fuel BC, even under differing ventilation conditions. The scented candle event (event 3) induced increases in both biomass and fossil-related BC, possibly due to incomplete combustion or the candle’s complex chemical composition. Vacuum cleaning (event 18) led to only a modest increase in biomass BC, suggesting limited resuspension of particulate matter. In contrast, the neighbor’s odor event (unplanned event 19) produced one of the sharpest peaks in fossil fuel BC across the monitoring period, indicating strong infiltration through an opened door from nearby outdoor combustion. A similarly high peak was observed the following day (30 October), although no specific event was detected at that time; the similarity in intensity and profile suggests that it may have originated from a comparable nearby combustion source. All three electric heater events showed small but noticeable increases, particularly in fossil fuel BC, likely due to trace emissions or associated ventilation patterns.

The position of event timing with the fossil fuel and biomass burning component of the BC trends in this figure provides an overview of how real-life activities and environmental conditions impact indoor air pollution in residential settings. It visually supports the hypothesis that combustion-related behaviors are among the most significant drivers of indoor BC, while other activities may have negligible or context-dependent influence. This graphical representation serves as a foundational reference for the more rigorous quantitative comparisons presented in subsequent sections, including event-level statistical testing and concentration profiling. For each event, BC concentrations (biomass burning and fossil fuel) were analyzed before, during, and after the intervention, and evaluated using one-way ANOVA and Kruskal–Wallis tests to assess statistical significance (Table 3). Due to the distances in the events’ timing, all are considered independent of each other.

Events involving combustion or open ventilation pathways (e.g., scented candle, gas burner, or doors open to the garden or hallway) often showed highly significant changes in biomass BC concentrations, with *p*-values below 0.001 in both ANOVA and Kruskal–Wallis tests. Notably, human presence and physical activity (e.g., rowing) also influenced biomass BC, suggesting resuspension or indirect infiltration effects. In contrast, fossil fuel BC responses were more pronounced during short-term combustion sources such as the gas burner (open and closed door), neighbor burning odor, and electric heater usage, several of which exhibited highly significant increases (*p* < 0.001). Interestingly, while some events like the scented candle or diffusers triggered significant responses in both BC types, others were more selective; for instance, exercise and open door ventilation primarily influenced biomass BC, whereas fossil BC was minimally affected. Conversely, vacuum cleaning, typically considered a resuspension source, had no measurable impact on biomass BC but showed a significant increase in fossil BC. These results show the emission profiles and source sensitivities of the two BC subtypes and underscore the importance of disaggregated analysis for accurate source attribution and exposure assessment in indoor air quality research.

**Figure 4:**
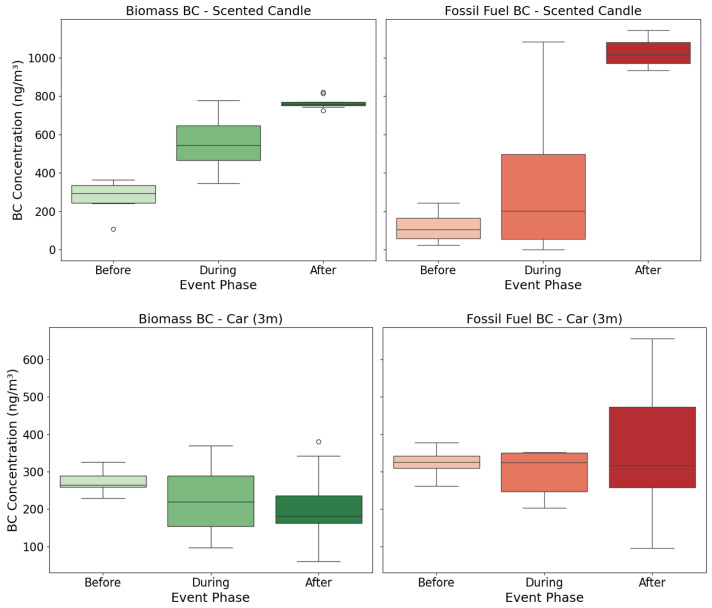
Changes in indoor biomass BC (green) and fossil fuel BC (red) concentrations before (lighter shades), during, and after (darker shades) selected combustion and infiltration events. Outliers are shown as hollow circles.

To better understand how specific household and environmental activities affect indoor BC concentrations, we analyzed two illustrative events using a pre/post-experimental design. For each, BC values were grouped into 15 min intervals before, during, and after the event. These groups were compared using boxplots (Figure 4) and the results were discussed in the context of ventilation, proximity to emission sources, and combustion type.

During the scented candle event, biomass-derived BC exhibited a consistent increase from the event period and further into the post-event phase, indicating that scented candles are a substantial source of biomass BC indoors. Interestingly, fossil fuel BC also increased, particularly after the event, suggesting either secondary emissions or inadequate post-combustion ventilation. In contrast, the car (3 m) event showed minimal change in biomass BC, consistent with expectations, but a notable rise in fossil fuel BC was observed after the event, reflecting short-term infiltration of combustion-related particles from the idling vehicle. The distributions of BC concentrations during the events were often more variable than those before or after. This increased variability likely reflects a combination of transient emission spikes, differences in source intensity, and dynamic airflow conditions (e.g., door/window positioning, or air mixing). For instance, the wide interquartile ranges and presence of outliers during the scented candle event suggest unstable dispersion of combustion particles and localized accumulation. In contrast, post-event concentrations for the car exposure case were consistently elevated, potentially due to delayed infiltration from outdoors or recirculation patterns near the sensor. These findings explain the value of separating BC by source to better understand indoor exposure dynamics and to connect specific activities contributing to biomass and fossil fuel PM.

Although the World Health Organization has not established a specific air quality guideline value for BC, it strongly recommends keeping BC concentrations as low as possible due to its carcinogenic properties and its role as a short-lived climate pollutant. According to the WHO Global Air Quality Guidelines, annual mean ambient BC concentrations in urban background settings typically range from 1.0 to 2.0 µg/m^3^, and significant health effects have been observed in epidemiological studies at mean BC levels as low as 1.08–1.15 µg/m^3^ [4]. In this study, several daily indoor mean concentrations approached or exceeded these levels, with the highest daily mean biomass BC reaching 1053.1 ng/m^3^ (1.05 µg/m^3^) and fossil fuel BC reaching 880.3 ng/m^3^ (0.88 µg/m^3^). Furthermore, peak 1 min values exceeded 5 µg/m^3^, highlighting potential short-term exposure spikes far above levels associated with adverse health outcomes in the literature. These findings revealed the health significance of indoor BC emissions, even in residential settings, and support strategies for reducing indoor exposure to combustion-related particles in line with WHO’s risk reduction approach.

## 4. Conclusions

This study provides a detailed, high-resolution assessment of indoor BC concentrations (biomass burning and fossil fuel components) in a residential setting over a 24-day period, capturing real-world variability driven by occupant behavior, combustion-related activities, and ventilation dynamics. By synchronizing minute-level BC measurements with a comprehensive log of timestamped experimental and natural events, including candle burning, cooking, heating, human presence, window/door usage, and outdoor traffic, we were able to isolate the specific influence of each source on indoor BC levels. Temporal analysis revealed daily and hourly variability in total BC, driven by both recurring indoor activities and episodic emission events. Through visual alignment of experimental activities with BC trends and statistical testing, we demonstrate that real-life indoor behaviors and environmental interactions have an influence on BC subtypes. Combustion-related activities, such as candle burning, gas burner use, and nearby combustion sources, were among the most influential, often causing statistically significant spikes in both biomass burning and fossil fuel BC concentrations. Ventilation-related events (e.g., door opening) typically elevated biomass BC, indicating the infiltration of outdoor air. At the same time, short-term mechanical sources like electric heaters and vehicle proximity were more associated with fossil-derived BC increases. In contrast, activities such as human presence or exercise had minor or inconsistent impacts, emphasizing the importance of source type and proximity.

Disaggregating BC into biomass burning and fossil fuel components revealed behavioral differences that would be masked when using total BC alone, enabling a more precise understanding of indoor exposure dynamics. The combined use of time-aligned visualization and event-level statistical testing proved effective in identifying which activities pose the greatest risk to indoor air quality. In summary, the study reveals the central role of combustion sources in indoor BC levels and establishes a framework for event-based pollutant profiling using synchronized sensor networks. While this study offers detailed insights, its findings are based on a single household, which may limit generalizability to other settings. However, due to the demanding nature of high-resolution, multi-sensor monitoring, this approach serves as a methodological foundation. Also, the current study provides detailed short-term information about BC behavior but does not show long-term seasonal variability. Future work should address this by extending monitoring across multiple seasons, diverse building types, and additional pollutants to assess how changes in heating practices, ventilation habits, and outdoor conditions influence indoor BC composition and concentrations.

## Figures and Tables

**Table 1:** General statistics for monitored period, daily values; total BC (TBC), biomass burning (BB), fossil fuel (FF), nan indicates missing value.

Date	Mean (TBC)	Min (TBC)	Max (TBC)	Mean (BB)	Mean (FF)
11 October 2024	884.43	94	1753	363.83	517.42
12 October 2024	518.8	63	1784	417.17	101.04
13 October 2024	356.62	48	1037	174.72	181.89
14 October 2024	558.02	84	1228	281.01	276.82
15 October 2024	799.07	60	3047	592.16	206.49
16 October 2024	398.84	152	692	288.84	109.8
17 October 2024	576.44	78	994	509.53	66.91
18 October 2024	612.88	337	801	559.68	53.19
19 October 2024	604.58	195	812	491.2	113.38
20 October 2024	532.97	84	1904	360.89	171.57
21 October 2024	1347.43	558	5430	779.39	568.04
22 October 2024	nan	nan	nan	nan	nan
23 October 2024	957.78	696	2964	691.57	266.22
24 October 2024	1009.46	561	1433	1009.46	0
25 October 2024	817.93	511	1085	754.96	62.98
26 October 2024	593.08	407	797	280.28	312.79
27 October 2024	718.3	430	903	450.99	267.31
28 October 2024	681.64	334	1026	520.16	161.48
29 October 2024	1111.63	157	3900	623.39	488.23
30 October 2024	1933.37	881	5133	1053.09	880.28
31 October 2024	1547.11	930	2833	892.46	654.65
1 November 2024	579.33	116	994	230.07	349.1
2 November 2024	729.32	31	2151	311.93	416.95
3 November 2024	716.84	14	5083	336.16	379.34

**Table 2:** Statistics for the time of the day; total BC (TBC), biomass burning (BB), fossil fuel (FF).

Metric	Day	Late Night	Night
BB BC Mean	441.73	662.22	698.43
BB BC Min	0	0	0
BB BC Max	1654	1736	1666
FF BC Mean	333.6	124.02	200.27
FF BC Min	0	0	0
FF BC Max	5133	561.38	929
TBC Mean	775.72	786.25	898.71
TBC Min	14	152	213
TBC Max	5430	1766	1833

**Table 3:** Results of ANOVA and Kruskal–Wallis tests (with *p* < 0.05 marked in red) for BC from biomass burning (BB) and fossil fuel (FF).

Event	ANOVA, *p* (BB)	Kruskal, *p* (BB)	ANOVA, *p* (FF)	Kruskal, *p* (FF)
Candle Burning (Closed Door 1)	0.280	0.218	0.073	0.057
Candle Burning (Closed Door 2)	0.852	0.884	0.885	0.721
Scented Candle	0.000	0.000	0.000	0.000
Candle Burning (Open Door)	0.001	0.000	0.028	0.002
Gas Burner (Closed Door)	0.222	0.317	0.018	0.023
Gas Burner (Open Door)	0.000	0.000	0.000	0.000
Human Presence (1)	0.007	0.010	0.000	0.001
Human Presence (2)	0.000	0.000	0.000	0.000
Exercise Rowing (Closed Door)	0.000	0.000	0.697	0.867
Exercise Rowing (Open Door)	0.000	0.000	0.192	0.142
Door Open to Garden	0.000	0.000	0.566	0.381
Door Open to Hallway	0.000	0.000	0.359	0.418
Doors Open (Garden + Hallway)	0.000	0.000	0.192	0.064
Car Running (3 m from door)	0.141	0.085	0.000	0.003
Car Running (5 m from door)	0.017	0.037	0.971	0.983
Diffuser (Water)	0.011	0.023	0.000	0.001
Diffuser (Oil)	0.001	0.001	0.000	0.000
Vacuum Cleaning (Windows Closed)	0.893	0.922	0.000	0.000
Neighbor Burning Odor	0.000	0.009	0.000	0.000
Tar Burning (Unplanned Event)	0.821	0.737	0.278	0.295
Electric Heater (1)	0.000	0.000	0.073	0.057
Electric Heater (2)	0.990	0.939	0.885	0.721
Electric Heater (3)	0.143	0.061	0.000	0.000
Central Heating (Radiator)	0.000	0.000	0.028	0.002
Aquarium Pump	0.383	0.614	0.018	0.023

## Data Availability

The dataset supporting the findings of this study is publicly available at https://doi.org/10.5281/zenodo.15224267.
